# Study on the Influence and Mechanism of Mineral Admixtures and Fibers on Frost Resistance of Slag–Yellow River Sediment Geopolymers

**DOI:** 10.3390/nano15131051

**Published:** 2025-07-06

**Authors:** Ge Zhang, Huawei Shi, Kunpeng Li, Jialing Li, Enhui Jiang, Chengfang Yuan, Chen Chen

**Affiliations:** 1 Yellow River Institute of Hydraulic Research, Yellow River Water Conservancy Commission, Zhengzhou 450003, China; gezhangyrihr@163.com (G.Z.); 15538352232@163.com (H.S.); 15617633649@163.com (C.C.); 2 Key Laboratory of Lower Yellow River Channel and Estuary Regulation, Ministry of Water Resources, Zhengzhou 450003, China; 3 Yellow River Laboratory, Zhengzhou 450003, China; 4 School of Water Conservancy and Transportation, Zhengzhou University, Zhengzhou 450001, China; jialingli1120@163.com; 5 College of Civil Engineering, Zhengzhou University, Zhengzhou 450001, China; chengfang1102@zzu.edu.cn

**Keywords:** Yellow River sediment (YRS), geopolymer composite, freeze-thaw cycle, microscopic mechanism

## Abstract

To address the demands for resource utilization of Yellow River sediment and the durability requirements of engineering materials in cold regions, this study systematically investigates the mechanisms affecting the frost resistance of slag-Yellow River sediment geopolymers through the incorporation of mineral admixtures (silica fume and metakaolin) and fibers (steel fiber and PVA fiber). Through 400 freeze-thaw cycles combined with microscopic characterization techniques such as SEM, XRD, and MIP, the results indicate that the group with 20% silica fume content (SF20) exhibited optimal frost resistance, showing a 19.9% increase in compressive strength after 400 freeze-thaw cycles. The high pozzolanic reactivity of SiO_2_ in SF20 promoted continuous secondary gel formation, producing low C/S ratio C-(A)-S-H gels and increasing the gel pore content from 24% to 27%, thereby refining the pore structure. Due to their high elastic deformation capacity (6.5% elongation rate), PVA fibers effectively mitigate frost heave stress. At the same dosage, the compressive strength loss rate (6.18%) and splitting tensile strength loss rate (21.79%) of the PVA fiber-reinforced group were significantly lower than those of the steel fiber-reinforced group (9.03% and 27.81%, respectively). During the freeze-thaw process, the matrix pore structure exhibited a typical two-stage evolution characteristic of “refinement followed by coarsening”: In the initial stage (0–100 cycles), secondary hydration products from mineral admixtures filled pores, reducing the proportion of macropores by 5–7% and enhancing matrix densification; In the later stage (100–400 cycles), due to frost heave pressure and differences in thermal expansion coefficients between matrix phases (e.g., C-(A)-S-H gel and fibers), interfacial microcracks propagated, causing the proportion of macropores to increase back to 35–37%. This study reveals the synergistic interaction between mineral admixtures and fibers in enhancing freeze–thaw performance. It provides theoretical support for the high-value application of Yellow River sediment in F400-grade geopolymer composites. The findings have significant implications for infrastructure in cold regions, including subgrade materials, hydraulic structures, and related engineering applications.

## 1. Introduction

Geopolymer (also known as alkali-excited cementitious materials) is characterized by fast setting speed [1,2]. high-temperature resistance [3,4,5], and corrosion resistance [6,7,8]. Geopolymer can be prepared without the need for calcination, offering great potential to reduce carbon emissions by replacing cement [9,10,11,12]. It has been applied in certain fields, such as civil engineering, rapid emergency repair, grouting materials, and heavy metal solidification [13]. To further enhance its performance, the incorporation of mineral admixtures has become an effective optimization strategy. Similar to conventional concrete, geopolymer materials suffer from high brittleness [14,15] and low tensile strength [16,17], which limits their widespread application [18,19]. Incorporating fibers into geopolymer materials can significantly enhance their engineering properties.

Lee et al. [20] have found that PET fibers have advantages such as hydrophilicity and dimple formation on the surface of the treated fiber, improving the bond strength property of the fiber matrix. These advantages compensate for the strength reduction due to weight loss in an alkaline environment. Lee et al. [21] investigated slag-based alkali-activated mortar-reinforced geopolymers with oiled PVAs and demonstrated the feasibility of achieving a tensile strain of up to 4.7%. Natali et al. [22] modified some properties of alkali-activated ladle slag at 7 d by employing PVAs, and the enhancement in ductility after the first crack load was obvious. Zhang et al. [23,24] investigated the behavior of the short PVA-reinforced fly ash-metakaolin geopolymer boards at 28 d. Their conclusion demonstrated that the incorporation of high-volume PVAs changed the impact failure mode of geopolymer boards from a brittle to ductile pattern. Akturk et al. [25] demonstrated that steel fiber or polypropylene fiber addition improved the flexural strength of sodium carbonate-activated slag mortars.

Frost resistance is a crucial aspect of cementitious materials’ durability [26]. Baomin Wang [27] studied the effects of alkali dosage, slag content, and curing age on the compressive strength of artificial flood-prevention stone. The frost resistance of the artificial flood-prevention stone at the age of 90 days was evaluated by the freeze-thaw cycle method. The results demonstrate that the frost resistance meets the requirement of 10 freeze-thaw cycles. Song Yingbin et al. [28] developed artificial flood control stone materials through alkali-activated resource utilization of Yellow River sediment, and investigated their frost resistance. The results demonstrated that after 40 freeze-thaw cycles, the specimens exhibited missing edges, significantly increased surface roughness, obvious fine aggregate attachment on the surface, severe mortar peeling, and embrittlement. Wang Zekun [29] conducted research on the preparation of unfired bricks using Yellow River sediment and investigated their frost resistance. The study revealed that after 20 freeze-thaw cycles, the mass loss rate of specimens generally exceeded 5%, while the compressive strength loss rate surpassed 50%. Ali Raza [30] evaluates the impact of different Yellow River sand replacement percentages (0, 25, 50, 75, and 100%) on ECC mechanical and microstructural properties under freeze-thaw conditions. Research indicates that frost resistance reaches its optimum when the Yellow River sand replacement ratio is 25%.

The plateau regions in the middle-upper Yellow River basin and seasonal frozen soil zones in the middle-lower reaches are extensively affected by freeze-thaw cycle damage, particularly in severely cold winter areas such as the Inner Mongolia Hetao Plain and Loess Plateau (with extreme temperatures reaching −30 °C), where annual freeze-thaw cycles exceed 50 times, posing significant challenges to engineering material durability. However, research on the frost resistance of Yellow River sediment geopolymer cementitious materials is limited. Therefore, systematic studies need to be conducted to elucidate the durability mechanisms of eco-friendly geopolymer materials derived from Yellow River sediments under freeze-thaw conditions. Such research would not only provide theoretical support for optimizing the design of construction materials in cold regions, but its findings could also be directly applied to critical infrastructure projects, including road foundations and underground engineering backfills in permafrost areas of the Yellow River basin, offering innovative solutions to address durability issues of engineering materials in these extreme environments.

## 2. Materials and Methods

### 2.1. Raw Material and Mixed Proportion

#### 2.1.1. Raw Material

The primary raw materials utilized in this study included yellow river silt (YRS), granulated blast furnace slag, silica fume (SF), metakaolin (MK), sodium hydroxide (NaOH), and water glass. The YRS was sourced from the Xixiayuan Reservoir in Henan Province and underwent oven-drying due to its initially moist state. After drying, its median particle size was measured at 0.20 mm. The slag had a median particle size of 10.30 μm and demonstrated a hydraulic coefficient of 2.18, an activity index of 0.47, and an alkalinity ratio of 1.17. The silica fume used possessed a specific surface area between 18,240 and 20,880 m^2^/kg, a density of 2.226 g/cm^3^, and an average particle size of 15.78 μm, with SiO_2_ content exceeding 92%. The metakaolin employed was highly reactive, exhibiting 7-day and 28-day activity indices of 115% and 118%, respectively, and had a median particle size of 5.06 μm. The chemical compositions of YRS, MK, slag, and SF were determined using X-ray fluorescence (XRF), with results detailed in Table 1. Two types of reinforcing fibers were incorporated: short, fine copper-coated steel fibers and short-cut polyvinyl alcohol (PVA) fibers. Their key performance indicators are provided in Table 2. Liquid sodium silicate (SS), supplied by Shandong Yourui Chemical Co., Ltd. (Weifang, China), was used as the alkali activator. Its modulus was adjusted to 1.2 using pure NaOH, and its properties are summarized in Table 3. Tap water served as the mixing medium throughout all experimental procedures.

#### 2.1.2. Mixed Proportion

Table 4 presents the mix proportions of the slag–Yellow River sediment-based geopolymer for a durability test. Prior to mixing, the pre-measured sodium hydroxide was completely dissolved in the sodium silicate solution to form the alkali activator. The blending process was carried out using a uniaxial horizontal concrete mixer. Initially, YRS and the mineral additives were added to the mixer and stirred for 180 s to ensure uniform distribution. The fibers were incorporated into the mixer, and the stirring continued for an additional 240 s. Subsequently, water was introduced into the mixer, and the mixture was stirred for 180 s. The water glass was added, and the mixture was stirred for 120 s. Immediately following this, the mixture was promptly poured into molds and then subjected to consolidation on a vibration table for a period ranging between 60 and 90 s. The molded specimens are covered for curing and then placed indoors for 1 day before demolding. After demolding, the specimens are placed in a standard curing room (20 °C ± 2 °C and RH > 95%) to be cured until 24 days of age. Subsequently, they are immersed in water at (20 ± 2) °C for 4 days before being removed for testing. The mix proportions are presented in Table 4, respectively.

### 2.2. Experimental Method

In this experiment, the effects of SF, MK, ST, and PVA on the frost resistance, characteristic products, and microstructural property tests of slag-yellow river sediment-based geopolymers were analyzed by the setting time, compressive and splitting tensile strength, hydration heat, thermogravimetric analysis, X-ray diffraction (XRD) analysis, porosity test (MIP), and scanning electron microscopy (SEM) test. Table 5 shows the test grouping, including the size and number of each test and specimen.

#### 2.2.1. Frost Resistance Test

The freeze-thaw resistance test is conducted using the rapid freezing method outlined in the standard GB/T 50082-2024 [31]. A total of 400 freeze-thaw cycles is performed. The specimens’ mass loss rate and dynamic elastic modulus are measured every 25 cycles, while compressive strength and splitting tensile strength are assessed every 50 cycles. The evaluation of freeze-thaw resistance employs two types of specimens: ① 100 mm × 100 mm × 400 mm, used to determine the dynamic elastic modulus and mass loss rate after freeze-thaw cycles; ② 100 mm × 100 mm × 100 mm, used to assess compressive strength and splitting tensile strength after freeze-thaw cycles.

#### 2.2.2. X-Ray Diffraction Analysis

Using the Japanese Physical X-ray diffractometer (Shimadzu Corporation, Kyoto, Japan), immerse the sample in anhydrous ethanol for more than 7 days to terminate the reaction, then take out the sample and put it into a vacuum drying oven for drying. After drying, grind the sample, pass it through a 200-mesh sieve, and put it into the glass groove for testing. The sampling interval is 0.04° (2θ), the sampling speed is 1°/min, and the scanning angle range is 5–70° (2θ).

#### 2.2.3. Thermogravimetric Analysis

A ZCT-B simultaneous thermal analyzer (Beijing Jingyi Hitech instrument Co., Ltd., Beijing, China) was employed. The sample preparation method is the same as XRD, taking about 20 mg of the sample to be tested, and the heating range is 30~1000 °C, the heating rate was controlled at 10 °C/min. The DTA curves for each group of samples were obtained.

#### 2.2.4. Porosity Test

The porosity and pore size distribution of the hardened paste were analyzed using the mercury intrusion method. Small-size paste specimens were prepared for standard curing (20 °C ± 2 °C, RH > 95%). After curing for 28 days, the hydrated samples were broken using pliers for testing. The porosity was measured using a Poremaster-33T automatic mercury porosimeter (Anton Paar Quanta Tec Inc., Boynton Beach, FL, American) with an aperture measurement range of 3.5 nm to 360,000 nm [32,33].

#### 2.2.5. Scanning Electron Microscopy Test

The microstructure of the cement paste samples was observed using a Sigma 300 field emission environmental scanning electron microscope (Carl Zeiss AG, Oberkochen, Germany). After curing for 28 days, the samples were broken with pliers, immersed in anhydrous ethanol for more than 7 days to terminate the reaction, and then placed in a vacuum drying oven for drying and the test was conducted after the drying process was completed [34].

## 3. Experiment Results and Analysis

### 3.1. Mass Loss Rate and Relative Dynamic Elastic Modulus

**Figure 1:**
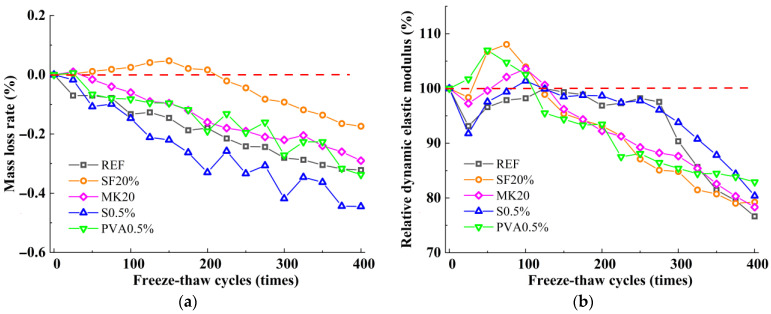
Mass loss rate and relative dynamic elastic modulus. (**a**) Mass loss rate and (**b**) relative dynamic elastic modulus.

Figure 1 illustrates the variation patterns of mass loss rate and relative dynamic elastic modulus of the slag-Yellow River sediment geopolymer under freeze-thaw cycles. As shown in Figure 1a, with increasing freeze-thaw cycles, the mass loss rate of SF20 exhibits two-stage characteristics: during the initial freeze-thaw stage (0–150 cycles), the mass loss rate increases slightly, reaching 0.05% after 150 cycles. With a further increase in freeze-thaw cycles, the mass loss rate commences a downward trend, ultimately attaining −0.17% after 400 freeze-thaw cycles. Unlike SF20, the mass loss rates of the reference group, MK20, ST0.5%, and PVA0.5% decreased gradually with freeze-thaw cycles, and after 400 freeze-thaw cycles, the mass loss rates of the reference group and MK20 were −0.32% and −0.29%, respectively. ST0.5 and PVA0.5 mass loss rates show significant fluctuations compared to the fiber-free control mix, although they show an overall decreasing trend. After 400 cycles, their mass loss rates attain values of −0.44% and −0.34%, respectively.

Figure 1b illustrates the relative dynamic elastic modulus variation patterns in the slag-Yellow River sediment geopolymer under freeze-thaw cycles. The figure shows that PVA0.5 demonstrates a two-stage evolution pattern characterized by initial strengthening followed by progressive deterioration. In contrast, the splitting tensile strength evolution of the reference group, SF20, MK20, and ST0.5, can be systematically categorized into three distinct stages: the initial deterioration phase, the strengthening phase, and the terminal deterioration phase. The relative dynamic elastic modulus of PVA0.5 reaches a maximum value of 106.98% after 50 freeze-thaw cycles. This modulus initiates a progressive decline with continued cycling, ultimately decreasing to 82.91% following 400 freeze-thaw cycles. The reference groups, SF20, ST0.5%, and MK20, exhibit an initial reduction in relative dynamic elastic modulus to 93.13%, 98.36%, 91.78%, and 97.26%, respectively, after 50 freeze-thaw cycles. With the increase in the number of freeze-thaw cycles, the relative dynamic modulus of elasticity shows a certain degree of increase; the reference group and SF20 reach maximum values of 100.00% and 108.06% following 125 and 75 cycles, and the ST0.5% and MK20 achieve maxima of 101.39% and 103.60% at 100 cycles. Subsequently, with the continuation of freeze-thaw cycles, all the mix ratios showed a decreasing trend, and the relative dynamic modulus of elasticity of the benchmark group, SF20, ST0.5%, and MK20 decreased to 75.64%, 79.26%, 80.39%, and 78.32%, respectively, after 400 cycles of freeze-thaw cycles. According to the evaluation requirements of the fast-freezing method for concrete, all formulated slag-Yellow River sediment geopolymers demonstrate exceptional frost resistance, uniformly attaining the F400 durability classification.

### 3.2. Compressive Strength

**Figure 2:**
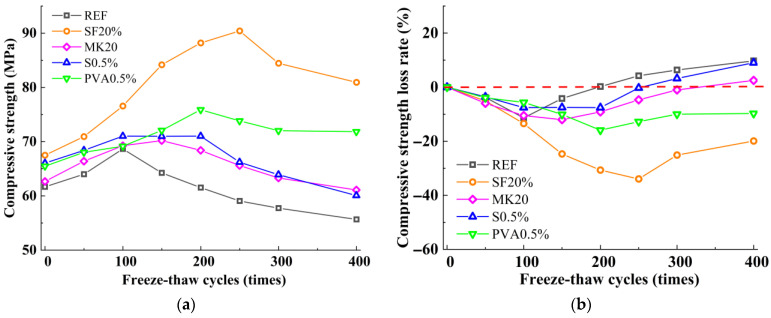
The variation in compressive strength of Slag-Yellow River Sediment geopolymers under freeze-thaw cycles. (**a**) Compressive strength; (**b**) compressive strength loss rate.

Figure 2a illustrates the variation patterns of compressive strength in the slag-Yellow River sediment geopolymer under freeze-thaw cycles, while Figure 2b delineates the corresponding compressive strength loss rate evolution derived from the data in Figure 2a. As shown in Figure 2a, with the increase in freeze-thaw cycles, the compressive strength of all mix proportions initially increases and then decreases, demonstrating a mechanical response pattern of first strengthening and then deterioration. However, significant strength variations exist among different mix proportions. Under freeze-thaw cycles, the reference group exhibited a strengthening phase during 0–150 cycles. After 150 freeze-thaw cycles, the compressive strength reached its maximum value of 64.25 MPa, representing an 11.33% increase compared to the initial strength. This enhancement is primarily attributed to the temperature alternation effects of freeze-thaw cycles, which promoted continuous hydration reactions of slag particles, generating more C-(A)-S-H gel phases to fill pores. With further increases in freeze-thaw cycles, the compressive strength begins to decline, primarily due to the temperature alternation effects that gradually develop and expand microcracks within the matrix, causing internal damage. After 400 freeze-thaw cycles, the compressive strength decreases to 55.67 MPa, with a compressive strength loss rate of 9.74%. Compared to the reference group, both MK20 and SF20 exhibited higher compressive strengths before freeze-thaw cycles, exceeding the reference group by 1.62% and 9.45%, respectively. As shown in Figure 2, under freeze-thaw cycles, MK20 and SF20 exhibited extended strengthening phases and more pronounced enhancement effects compared to the reference group. The compressive strengths of MK20 and SF20 reached their maximum values at 150 freeze-thaw cycles and 250 freeze-thaw cycles, respectively, representing increases of 12.02% and 33.94% compared to pre-freeze-thaw conditions. With further increases in freeze-thaw cycles, the compressive strengths of MK20 and SF20 began to decline. After 400 freeze-thaw cycles, their compressive strengths decreased to 61.10 MPa and 80.94 MPa, with strength loss rates of 2.51% and −19.90%, respectively. Compared to the reference group, ST0.5 and PVA0.5 also showed higher compressive strengths before freeze-thaw cycles, exceeding the reference group by 7.09% and 6.18%, respectively. Under freeze-thaw cycles, both ST0.5 and PVA0.5 reached their maximum values at 200 freeze-thaw cycles, with increases of 7.54% and 15.90% compared to pre-freeze-thaw conditions. As the number of cycles continued to rise, their compressive strengths declined. After 400 freeze-thaw cycles, the compressive strengths decreased to 60.08 MPa and 71.84 MPa; the compressive strength showed a decrease of 9.03% and an increase of 9.71%, respectively, compared to pre-freeze-thaw cycle levels.

### 3.3. Splitting Tensile Strength

**Figure 3:**
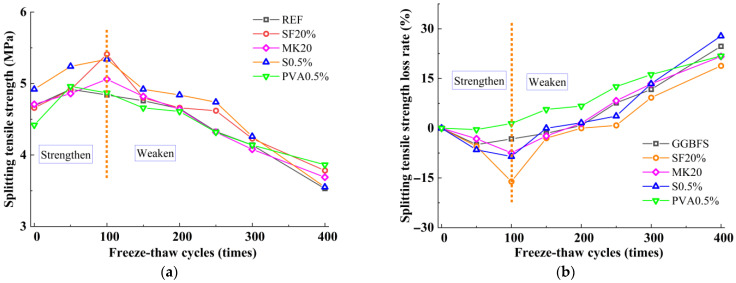
The variation in compressive strength of Slag-Yellow River Sediment geopolymers under freeze-thaw cycles. (**a**) Splitting tensile strength; (**b**) splitting tensile strength loss rate.

Figure 3a illustrates the variation in splitting tensile strength of slag-Yellow River sediment geopolymer under freeze-thaw cycles, while Figure 3b shows the corresponding splitting tensile strength loss rates calculated based on Figure 3a. As shown in Figure 3a, similar to the compressive strength trend, the splitting tensile strength of all mix proportions first increases and then decreases with the increase in freeze-thaw cycles, demonstrating a mechanical response pattern of first strengthening and then deterioration. However, significant strength variations are observed among different mix proportions.

Under freeze-thaw cycles, the reference group exhibited a strengthening phase during 0–50 cycles. When the freeze-thaw cycles reached 50 cycles, the splitting tensile strength attained its maximum value of 4.92 MPa, representing a 4.94% increase compared to the initial strength. With further increases in freeze-thaw cycles, the splitting tensile strength began to decline. After 400 freeze-thaw cycles, the splitting tensile strength decreased to 3.53 MPa, corresponding to a splitting tensile strength loss rate of 24.70%.

Compared to the reference group, MK20 and SF20 exhibited splitting tensile strengths comparable to the reference group before freeze-thaw cycles. Under freeze-thaw cycles, however, MK20 and SF20 demonstrated longer strengthening phases and more pronounced enhancement effects than the reference group. The splitting tensile strengths of MK20 and SF20 reached their maximum values at 100 freeze-thaw cycles, with increases of 7.43% and 16.09%, respectively, compared to pre-freeze-thaw conditions. With further increases in cycles, their splitting tensile strengths began to decline. After 400 freeze-thaw cycles, the strengths decreased to 3.69 MPa and 3.78 MPa, corresponding to splitting tensile strength loss rates of 21.66% and 18.81%, respectively. Compared to the reference group, ST0.5 and PVA0.5 showed higher splitting tensile strengths before freeze-thaw cycles, exceeding the reference group by 4.94% and 5.37%, respectively. Under freeze-thaw cycles, ST0.5 and PVA0.5 reached their maximum values at 100 freeze-thaw cycles and 50 freeze-thaw cycles, respectively, with increases of 8.54% and 0.40% compared to pre-freeze-thaw conditions. As the cycles continued, their splitting tensile strengths began to decline. After 400 freeze-thaw cycles, the strengths decreased to 3.55 MPa and 3.86 MPa, with loss rates of 27.81% and 21.79%, respectively. Due to the higher sensitivity of splitting tensile strength to internal crack distribution and interfacial conditions within the matrix, under the same number of freeze-thaw cycles, all mix proportions exhibited higher loss rates in splitting tensile strength compared to compressive strength, along with shorter durations of the strengthening phase than observed in compressive strength behavior.

In summary, under freeze-thaw cycles, the strength of the slag-Yellow River sediment geopolymer exhibits a distinct “strengthen-weaken” pattern; the early stage is dominated by repair-strengthening due to secondary hydration, and the later stage is dominated by damage due to temperature alternation effects. The addition of MK and SF enhances the mechanical properties of the slag-Yellow River sediment geopolymer under freeze-thaw cycles, though through different mechanisms. MK contributes reactive Al_2_O_3_ to form a 3D N-A-S-H network, increasing gel polymerization, while SF leverages its high specific surface area and reactivity to sustain secondary hydration during freeze-thaw exposure, producing low calcium-to-silica ratio C-(A)-S-H gel with superior chemical stability. Steel fibers, due to the large difference between the coefficient of thermal expansion and the matrix, therefore, under the action of temperature alternation, will produce uneven deformation, especially at the interface, which will produce cracks, reducing the adhesion between the fibers and the matrix and the densification of the matrix inside. In contrast, PVA fibers effectively alleviate frost heave stresses through high elastic deformability, demonstrating better performance than steel fibers.

### 3.4. Tension-Compression Ratio

**Figure 4:**
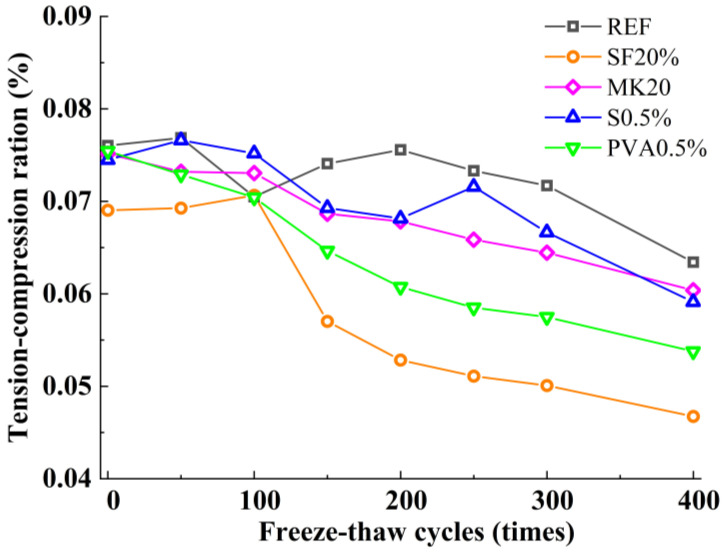
Tension-compression ratio.

Figure 4 illustrates the variation in the tension-compressive ratio of the slag-Yellow River sediment geopolymer under freeze-thaw cycles. As shown, all mix proportions exhibit a gradual decline in this ratio. This trend arises because the splitting tensile strength is more sensitive to internal crack distribution and interfacial conditions within the matrix. Consequently, under the same number of freeze-thaw cycles, the loss rate of split tensile strength was higher than the compressive strength for each mix ratio, and the duration of the strengthening section was shorter than the compressive strength. These combined effects lead to the observed reduction in the tensile-to-compressive strength ratio with increasing cycles. In comparison, the ratios for SF20 and PVA0.5 decline more significantly; this is due to the fact that SF20 and PVA0.5 are significantly more effective in strengthening compressive strength than split tensile strength.

### 3.5. Five-Dimensional Evaluation Diagram

**Figure 5:**
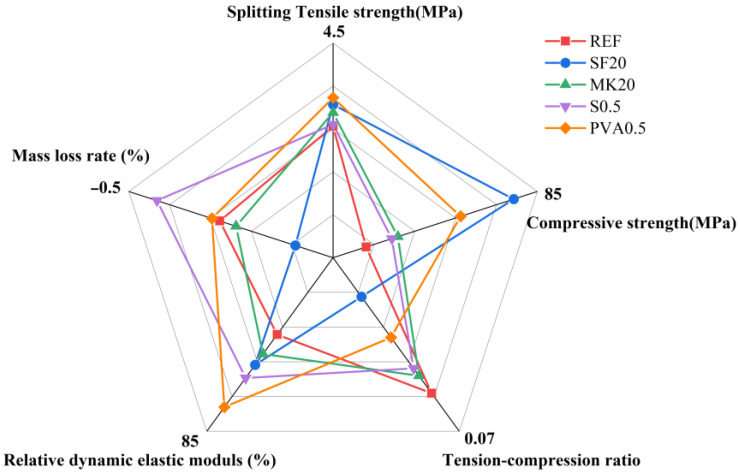
Five-dimensional evaluation diagram.

Based on the freeze-thaw cycle test results, this study selected five key indices for evaluation: compressive strength, splitting tensile strength, tension-compression ratio, mass loss rate, and relative dynamic elastic modulus, and conducted a comparative analysis using the five-dimensional assessment method [35]. The five-dimensional evaluation diagrams for slag-Yellow River sediment geopolymers with different mix proportions are presented in Figure 5. As shown in Figure 5, the SF20 mix demonstrates superior frost resistance compared to other proportions, exhibiting a 19.90% increase in compressive strength after 400 freeze-thaw cycles alongside the lowest splitting tensile strength loss rate while maintaining balanced performance in mass loss rate and relative dynamic elastic modulus. This is mainly due to the fact that, on the one hand, the fine particle size of silica fume enables a micro-aggregate effect that fills capillary pores and densifies the matrix, and on the other hand, the highly reactive SiO_2_ participates in secondary hydration reactions under freeze-thaw conditions to form dense C-(A)-S-H gel, which significantly strengthens the interfacial transition zone. The results of the five-dimensional evaluation confirm that the SF20 ratio is the most promising mix design to achieve the engineering application of geopolymers in severe cold regions.

## 4. Influence Mechanism Analysis

### 4.1. Characteristic Reaction Products

The existing research has shown that different types of fibers do not alter the categories of characteristic products in geopolymer cementitious materials [36]. Under freeze-thaw cycles using pure water as the medium in temperature-alternating environments, the characteristic products of the matrix are primarily influenced by the type and dosage of cementitious materials. Therefore, in Section 4.1 regarding characteristic reaction products, this study systematically investigates three sample groups: the reference group, SF20, and MK20.

**Figure 6:**
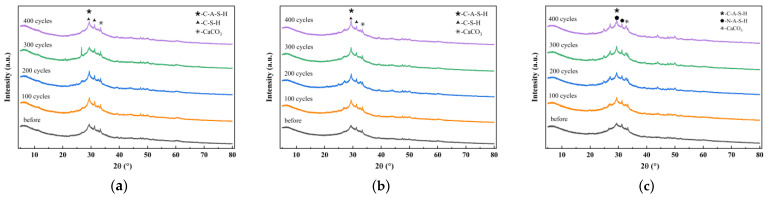
Comparative study of REF, SF20, and MK20 XRD patterns under different freeze-thaw cycles. (**a**) GGBFS; (**b**) SF20; (**c**) MK20.

Figure 6a–c present the XRD patterns of the reference group, SF20 and MK20, after different numbers of freeze-thaw cycles, respectively. To avoid the influence of impurities and minerals in the sediment on the products, the sample preparation involved using a paste sample with the sediment removed. As can be seen from the figures in Figure 6a–c, the phases in the groups were similarly composed of amorphous gel. The peaks observed between the diffraction angles of 20–40° are indicative of gels and CaCO_3_ in calcium-containing systems [37,38].

Figure 6a, b show that the C-(A)-S-H gels in the reference group and SF20 gradually expand with increasing freeze-thaw cycles. Unlike slag and silica fume, metakaolin, as a typical non-calcium aluminosilicate admixture, primarily generates C-A-S-H and N-A-S-H as the main reaction products, as illustrated in Figure 6c. The characteristic peak intensities of the gels progressively strengthen with prolonged freeze-thaw cycling, indicating that the mineral admixtures continue to undergo later-stage reactions under freeze-thaw conditions.

**Figure 7:**
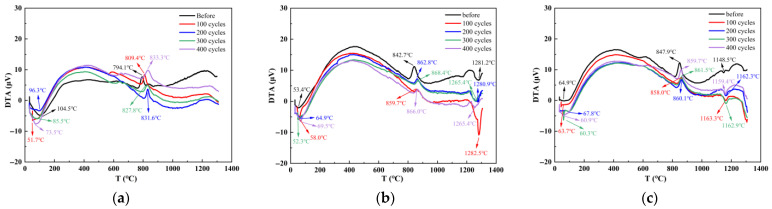
Comparative study of REF, SF20, and MK20 thermal analysis curves under different freeze-thaw cycles. (**a**) REF; (**b**) SF20; (**c**) MK20.

Figure 7a–c display the thermal analysis spectra of the reference group, SF20 and MK20, under different numbers of freeze-thaw cycles, respectively. The analysis results indicate that all three groups exhibit two distinct characteristic peaks at 60–100 °C and 700–800 °C. The heat absorption peak and the corresponding mass loss in the temperature interval from 60 to 100 °C are mainly attributed to the matrix pore structure and the removal of free and adsorbed water from the gel-like hydration products. The exothermic peak and mass loss observed in the 700–800 °C range originate from the dehydration and decomposition processes of gel-type products. With increasing freeze-thaw cycles, the intensities of these characteristic thermal analysis peaks in all three groups demonstrate a strengthening trend.

As shown in Figure 7a, after 0–400 freeze-thaw cycles, the low-temperature thermal decomposition temperatures of the reference group were measured at 104.5 °C, 51.7 °C, 96.3 °C, 85.5 °C, and 73.5 °C. In contrast, the high-temperature thermal decomposition temperatures were 794.1 °C, 809.4 °C, 831.6 °C, 827.8 °C, and 833.3 °C, respectively. The study revealed that the low-temperature decomposition temperatures were lower with progressive freeze-thaw cycling than before. In contrast, the high-temperature decomposition temperatures exceeded their pre-cycling values. This phenomenon can be attributed to the dual role of free water during freeze-thaw cycling. On one hand, water adsorbed on gel surfaces enhances thermal decomposition intensity in the 60–100 °C range. On the other hand, free water participates in the gel densification process, generating low calcium-to-silica ratio gels with improved thermal stability, thereby increasing the thermal decomposition temperatures. As shown in Figure 7b, after 0–400 freeze-thaw cycles, the low-temperature thermal decomposition temperatures of SF20 were measured at 53.4 °C, 58.0 °C, 64.9 °C, 52.3 °C, and 69.5 °C, while the high-temperature thermal decomposition temperatures reached 842.7 °C, 859.7 °C, 862.8 °C, 868.4 °C, and 866.0 °C, respectively. The study revealed that the low-temperature decomposition temperatures exhibited minor fluctuations with increasing freeze-thaw cycles, whereas all high-temperature decomposition temperatures exceeded their pre-cycling values. This behavior is attributed to the large specific surface area of silica fume, which had already adsorbed substantial free water before testing, resulting in smaller thermal decomposition temperature variations in the 60–100 °C range. Simultaneously, the free water participated in gel densification reactions, promoting the formation of low calcium-to-silica ratio gels, thereby similarly increasing the thermal decomposition temperatures. As shown in Figure 7c, after 0–400 freeze-thaw cycles, the low-temperature thermal decomposition temperatures of MK20 were measured at 64.9 °C, 63.7 °C, 67.8 °C, 60.3 °C, and 60.9 °C, while the high-temperature thermal decomposition temperatures reached 847.9 °C, 858.0 °C, 860.1 °C, 861.5 °C, and 859.7 °C, respectively. The study revealed that the low-temperature decomposition temperatures exhibited minor variations with increasing freeze-thaw cycles, whereas all high-temperature decomposition temperatures similarly exceeded their pre-cycling values. This phenomenon is attributed to the lamellar structure of metakaolin, which can adsorb substantial free water, resulting in limited thermal decomposition temperature differences in the 60–100 °C range. Simultaneously, the free water participates in the gel densification process, promoting the formation of low calcium-to-silica ratio gels with enhanced thermal stability, thereby similarly increasing the thermal decomposition temperatures.

In contrast to the reference group, SF20 and MK20 exhibit thermal decomposition peaks in the temperature ranges of 1200–1300 °C and 1100–1200 °C, respectively. There is a possible phase transition in the subsequent reaction involving silica powder and metakaolin under the effect of temperature alternation, transforming the more thermally stable cristobalite phase and mullite crystals, respectively.

### 4.2. Pore Structure

**Figure 8:**
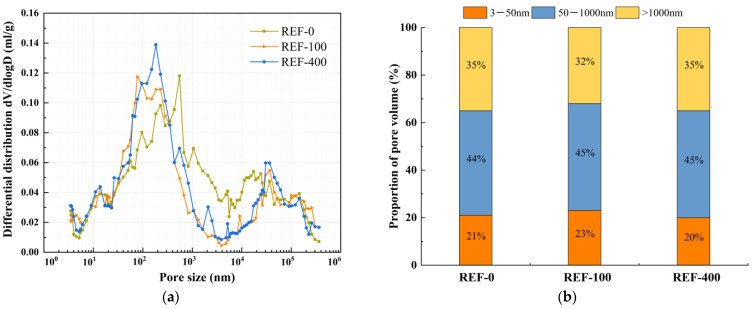
Comparative study of REF under different freeze-thaw cycles. (**a**) Relationship between dV/dlogD and pore size, (**b**) proportion of pore volume.

**Figure 9:**
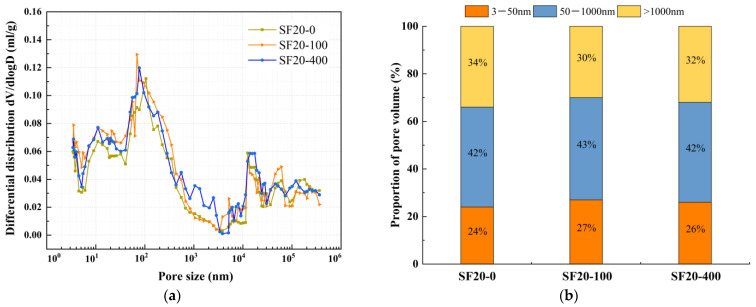
Comparative study of SF20 under different freeze-thaw cycles. (**a**) Relationship between dV/dlogD and pore size, (**b**) proportion of pore volume.

**Figure 10:**
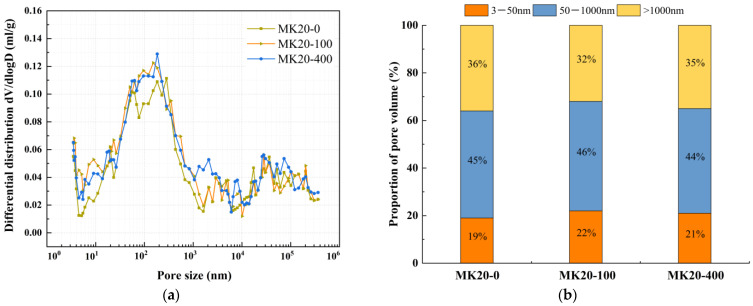
Comparative study of MK20 under different freeze-thaw cycles. (**a**) Relationship between dV/dlogD and pore size, (**b**) proportion of pore volume.

**Figure 11:**
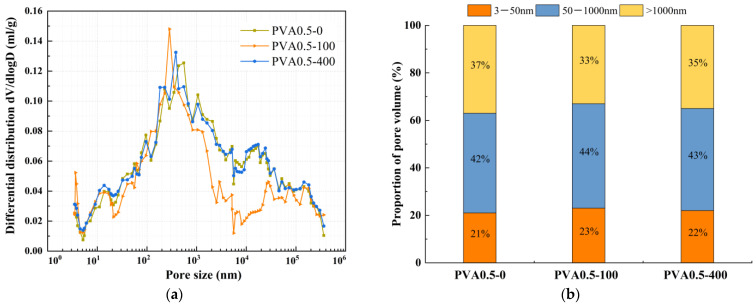
Comparative study of PVA0.5 under different freeze-thaw cycles. (**a**) Relationship between dV/dlogD and pore size, (**b**) proportion of pore volume.

**Figure 12:**
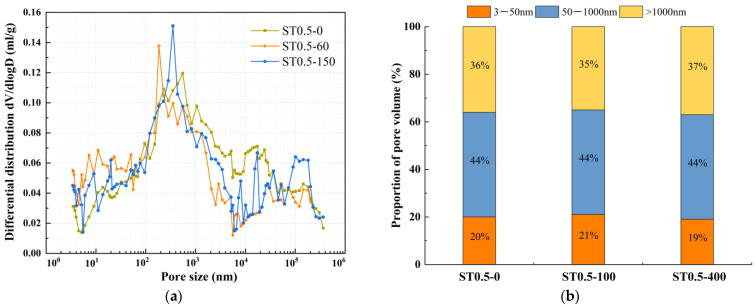
Comparative study of ST0.5 under different freeze-thaw cycles. (**a**) Relationship between dV/dlogD and pore size, (**b**) proportion of pore volume.

The effect of mineral admixtures and fibers on pore structure under different freeze-thaw cycles was selected for MIP experiments, as shown in Figure 8, Figure 9, Figure 10, Figure 11 and Figure 12. According to the results of the mercury injection test, in order to better study the relationship between pore-volume distribution and strength, the pore size is divided into three intervals: 3–50 nm, 50–1000 nm, and >1000 nm. The pore structure significantly affects the strength development of the matrix, and the mechanisms of different sizes of pores have obvious differences. Gel pores (<50 nm) are mainly distributed inside the C-S-H gel and between its particles. These pores positively affect the densification of the matrix [39]. In the later curing process, gel pores provide a channel for water migration and promote the continuous hydration of incompletely reacted mineral admixtures (such as slag) to produce C-(A)-S-H gel and other products, to refine the pore structure and improve the compactness of the matrix, which is conducive to the stable development of strength [40]. In contrast, macropores larger than 1000 nm are generally considered harmful pores, and their adverse effects are mainly reflected in two aspects. First, macropores tend to become stress concentration points when subjected to stress. According to Griffith’s fracture theory, the larger the pore size, the lower the stress required for critical crack growth, which will significantly reduce the bearing capacity of the material. Second, these macropores often connect to form a permeability network, mainly concentrated in the aggregate slurry interface (ITZ region). The porosity of this region is high, and the existence of macropores will further weaken the interfacial bonding performance, making the material preferentially break from the ITZ region when stressed, which will seriously affect the overall mechanical properties of the material [41].

The relationship between dV/dlogD and the pore size of REF under different freeze-thaw cycles is shown in Figure 8a. The pore volume of REF under different freeze-thaw cycles is shown in Figure 8b. As shown in Figure 8a, it can be observed that the most probable pore size of the reference group before freeze-thaw cycles is 151 nm, and 77 nm and 183 nm after 100 and 400 freeze-thaw cycles, respectively, which decreased and then increased with the increase in the number of freeze-thaw cycles. Meanwhile, Figure 8b reveals that with progressive freeze-thaw cycling, the gel pore proportion of the reference group first increased and then decreased, while the macropore proportion exhibited an inverse trend of initial reduction followed by expansion. The transitional pore proportion showed a slight increase throughout the process. After 100 freeze-thaw cycles, the gel pore proportion increased from 21% to 23%, accompanied by a decrease in macropore proportion from 35% to 32%, indicating pore structure refinement. This may be one of the main reasons for the increase in compressive strength and split tensile strength of the reference group, which indirectly confirms at the pore structure level that the subsequent reaction of the mineral admixtures occurs under freeze-thaw cycling and fills the voids of the matrix. As the freeze-thaw cycle continued, the percentage of macropores showed another increasing trend. The pore structure was gradually coarsened, and after 400 freeze-thaw cycles, the rate of gel pores decreased to 20%, and the percentage of macropores increased to 35%.

The relationship between dV/dlogD and the pore size of SF20 under different freeze-thaw cycles is shown in Figure 9a. The pore volume of SF20 under different freeze-thaw cycles is shown in Figure 9b. From Figure 9a, it can be observed that the most probable pore size of SF20 before freeze-thaw cycles is 105 nm, and 69 nm and 77 nm after 100 and 400 freeze-thaw cycles, respectively, which decreased and then increased with the increase in the number of freeze-thaw cycles. Meanwhile, Figure 9b reveals that with progressive freeze-thaw cycling, the gel pore proportion of SF20 first increased and then decreased. In contrast, the macropore proportion exhibited an inverse trend of initial reduction followed by expansion. The transitional pore proportion showed a slight increase throughout the process. After 100 freeze-thaw cycles, the gel pore proportion increased from 24% to 27%, accompanied by a decrease in macropore proportion from 34% to 30%, indicating pore structure refinement. This may be one of the main reasons for the increase in compressive strength and split tensile strength of SF20, which indirectly confirms at the pore structure level that the subsequent reaction of the silica powder as well as the mineral powder synergistically occurs under freeze-thaw cycling and fills the internal voids of the matrix. As the freeze-thaw cycles continued, the proportion of macropores exhibited an increasing trend once again. After 400 freeze-thaw cycles, the proportion of gel pores decreased to 26%, which was still higher than the pre-freeze-thaw level. This might partially explain why the compressive strength remained elevated compared to the initial state. Concurrently, under the persistent damaging effects of temperature fluctuations during freeze-thaw cycles, microcrack propagation caused by freeze-thaw damage led to an increase in macropores to 36%, surpassing the pre-freeze-thaw value. In contrast, the growth of macropores tends to induce stress concentration. Meanwhile, splitting tensile strength demonstrates higher sensitivity to internal crack distribution and interfacial characteristics within the matrix, which is one of the reasons why its splitting tensile strength shows a certain decline.

The relationship between dV/dlogD and the pore size of MK20 under different freeze-thaw cycles is shown in Figure 10a. The pore volume of MK20 under different freeze-thaw cycles is shown in Figure 10b. As shown in Figure 10a, it can be observed that the most probable pore size of MK20 before freeze-thaw cycles is 283 nm, and 77 nm and 183 nm after 100 and 400 freeze-thaw cycles, respectively, which decreased and then increased with the increase in the number of freeze-thaw cycles. Meanwhile, Figure 10b reveals that with progressive freeze-thaw cycling, the gel pore proportion of MK20 first increased and then decreased, while the macropore proportion exhibited an inverse trend of initial reduction followed by expansion. After 100 freeze-thaw cycles, the gel pore proportion increased from 19% to 22%, accompanied by a decrease in macropore proportion from 36% to 32%, indicating pore structure refinement. This may be one of the main reasons for the increase in compressive strength and split tensile strength of MK20, which indirectly confirms at the pore structure level that the subsequent reaction of the mineral powder and metakaolin occurs under freeze-thaw cycling and fills the voids of the matrix. As the freeze-thaw cycle continued, the percentage of macropores showed another increasing trend, and the pore structure gradually coarsened, and after 400 freeze-thaw cycles, the percentage of gel pores decreased to 21% and the percentage of macropores increased to 35%.

The relationship between dV/dlogD and the pore size of PVA0.5 under different freeze-thaw cycles is shown in Figure 11a. The pore volume of PVA0.5 under different freeze-thaw cycles is shown in Figure 11b. As shown in Figure 11a, it can be observed that the most probable pore size of PVA0.5 before freeze-thaw cycles is 433 nm, and 284 nm and 382 nm after 100 and 400 freeze-thaw cycles, respectively, which decreased and then increased with the increase in the number of freeze-thaw cycles. Meanwhile, Figure 11b reveals that with progressive freeze-thaw cycling, the gel pore proportion of PVA0.5 first increased and then decreased. In contrast, the macropore proportion exhibited an inverse trend of initial reduction followed by expansion. The transitional pore proportion showed a slight increase throughout the process. After 100 freeze-thaw cycles, the gel pore proportion increased from 21% to 23%, accompanied by a decrease in macropore proportion from 37% to 33%, indicating pore structure refinement. This may be one of the main reasons for the increase in compressive strength and split tensile strength of PVA0.5, which indirectly confirms at the pore structure level that the subsequent reaction of the mineral powder and metakaolin occurs under freeze-thaw cycling and fills the voids of the matrix. As the freeze-thaw cycle continued, the percentage of macropores showed another increasing trend, and the pore structure gradually coarsened. After 400 freeze-thaw cycles, the percentage of gel pores decreased to 22%, and the percentage of macropores increased to 35%.

The relationship between dV/dlogD and the pore size of ST0.5 under different freeze-thaw cycles is shown in Figure 12a. The pore volume of ST0.5 under different freeze-thaw cycles is shown in Figure 12b. As shown in Figure 12a, it can be observed that the most probable pore size of ST0.5 before freeze-thaw cycles is 551 nm, and 183 nm and 350 nm after 100 and 400 freeze-thaw cycles, respectively, which decreased and then increased with the increase in the number of freeze-thaw cycles. Meanwhile, Figure 12b reveals that with progressive freeze-thaw cycling, the gel pore proportion of ST0.5 first increased and then decreased, while the macropore proportion exhibited an inverse trend of initial reduction followed by expansion. After 100 freeze-thaw cycles, the gel pore proportion increased from 20% to 21%, followed by a macropore proportion decrease from 36% to 37%. This may be one of the main reasons for the increased compressive strength and split tensile strength of ST0.5. As the freeze-thaw cycle continued, the percentage of macropores showed an increasing trend again, and the pore structure gradually showed a coarsening effect, and after 400 freeze-thaw cycles, the percentage of gel pores decreased to 19% and the percentage of macropores increased to 37%. As the freeze-thaw cycle continued, the percentage of macropores showed an increasing trend again, and the pore structure gradually showed a coarsening effect, and after 400 freeze-thaw cycles, the percentage of gel pores decreased to 19%, and the percentage of macropores increased to 37%. Compared with PVA fiber, steel fiber has a large difference between the coefficient of thermal expansion and the matrix due to the thermal expansion coefficient. Therefore, under the effect of temperature alternation, steel fiber and the matrix will produce uneven deformation, especially at the interface. Cracks will be produced, which will reduce the adhesion between the fiber and the matrix and the densification inside the matrix, and the degree of pore structure coarsening will be more significant, which may be one of the reasons that, after 400 times of freeze-thaw cycling, the compressive and splitting tensile strength of ST0.5 The loss rate of ST0.5 was higher than that of PVA0.5.

### 4.3. Matrix Microstructure

**Figure 13:**
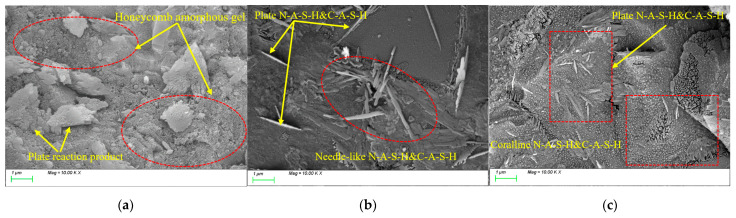
Microscopic image of REF under different freeze-thaw cycles. (**a**) before freeze-thaw cycles; (**b**) 100 freeze-thaw cycles; (**c**) 400 freeze-thaw cycles.

Figure 13a–c shows the microstructural morphology images of the matrix before freeze-thaw cycles, after 100 freeze-thaw cycles, and after 400 freeze-thaw cycles, respectively. It can be clearly observed from Figure 13a that, before freeze-thaw cycles, the characteristic products within the matrix exhibit two typical microstructural morphologies: one is a honeycomb-like amorphous gel composed of abundant pores, displaying a continuous porous network structure on its surface; the other consists of plate-like products with distinct crystalline boundaries and regular geometric shapes. These two types of products are interwoven within the matrix. After 100 freeze-thaw cycles, as shown in Figure 13b, compared to the initial state, the characteristic products exhibit significant growth trends with complete morphological development and clearly distinguishable outlines. This is evidenced by the formation of two typical gel products, plate-like and needle-like, mainly in the matrix. The plate-like products show a notable increase in size with sharp-edged boundaries, while the needle-like products display radial growth patterns. These gel products interconnect through mutual overlapping and interweaving, forming a continuous three-dimensional network structure. This network system, reinforced by the filling effect between adjacent gel phases, effectively refines the internal pore structure of the matrix, leading to a marked reduction in pore size at the microscale and more uniform pore distribution. Consequently, the structural compactness of the matrix is significantly enhanced. After 400 freeze-thaw cycles, as shown in Figure 13c, the gel products within the matrix exhibit distinct phase separation characteristics: one type manifests as coral-like amorphous gel with complex branching structures featuring rough surfaces and numerous pores; the other appears as plate-like gel with higher crystallinity, displaying clear grain boundaries and smooth surface morphology. Notably, significant microcrack networks with irregular radial distribution are observed both at the interface regions between the two gel phases and within the matrix. This damage pattern arises from two primary factors: sustained damage caused by ice-induced stresses during freeze-thaw cycles, and thermal stress accumulation resulting from differences in thermal expansion coefficients among various phase components under temperature alternation. Given that split tensile strength demonstrates higher sensitivity to crack distribution and interfacial conditions within the matrix, this microscale damage evolution significantly affects the degradation of matrix splitting tensile strength.

**Figure 14:**
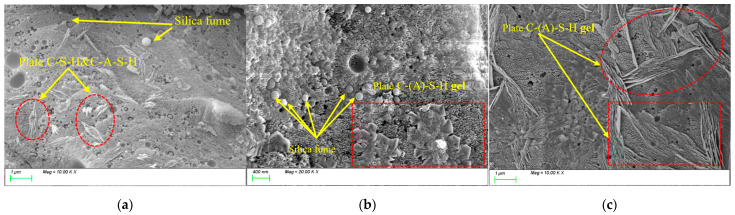
Microscopic image of SF20 under different freeze-thaw cycles. (**a**) before freeze-thaw cycles; (**b**) 100 freeze-thaw cycles; (**c**) 400 freeze-thaw cycles.

Figure 14 illustrates the evolution of microstructural morphology in SF20 specimens under varying numbers of freeze-thaw cycles. Figure 14a shows the SEM image before freeze-thaw cycles, where silica fume particles predominantly exhibit spherical or near-spherical morphology with smooth and flat surfaces and uniformly distributed particle sizes. Additionally, plate-like C-(A-)S-H gels can be observed randomly dispersed within the matrix. Figure 14b depicts the microstructure after 100 freeze-thaw cycles. While spherical silica fume particles remain discernible, their quantity and particle size exhibit significant reduction compared to the initial state. This indicates that a large amount of silica powder was involved in the subsequent reaction during the freeze-thaw processes, attributable to its ultrafine particle size and high specific surface area, which substantially enhance surface reactivity and promote conversion to plate-like C-(A-)S-H gels. Notably, the C-(A-)S-H gels in the matrix demonstrate distinct cluster-like interwoven structures. Such dense microstructural configurations constitute a critical factor contributing to the strength enhancement of the specimen. Figure 14c presents the microstructure after 400 freeze-thaw cycles. At this stage, intact silica fume particles are scarcely observable in the matrix. Instead, abundant plate-like products and gels are densely interconnected, forming a compact matrix structure. However, discrepancies in thermal expansion coefficients and mechanical properties between distinct phase products lead to interfacial defects during freeze-thaw cycles, primarily caused by ice-induced stresses and incompatible thermal deformation. These microstructural imperfections exert detrimental effects on the split tensile strength of the material.

**Figure 15:**
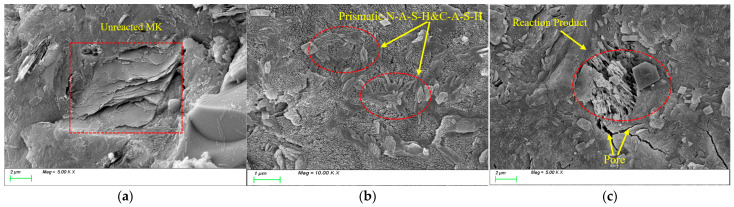
Microscopic image of MK-20 under different freeze-thaw cycles. (**a**) before freeze-thaw cycles; (**b**) 100 freeze-thaw cycles; (**c**) 400 freeze-thaw cycles.

Figure 15 illustrates the evolution of microstructural morphology in MK20 specimens under varying numbers of freeze-thaw cycles. Figure 15a shows the SEM image of the initial state, where a large number of unreacted metakaolin particles within the matrix can be clearly observed. These particles exhibit a typical lamellar morphology with smooth and flat surfaces, demonstrating an intercalated stacking configuration within the matrix. After 100 freeze-thaw cycles (Figure 15b), the microstructure undergoes significant changes: the lamellar metakaolin particles have almost disappeared. They are replaced by abundant prismatic C-A-S-H and N-A-S-H gel products. These products tightly interconnect to form a three-dimensional network structure, resulting in the matrix’s highly dense microstructural characteristic. However, when the freeze-thaw cycles reach 400 times (Figure 15c), although newly generated reaction products can still be observed, the matrix structure exhibits significant deterioration, and a discontinuous microcrack network has formed.

**Figure 16:**
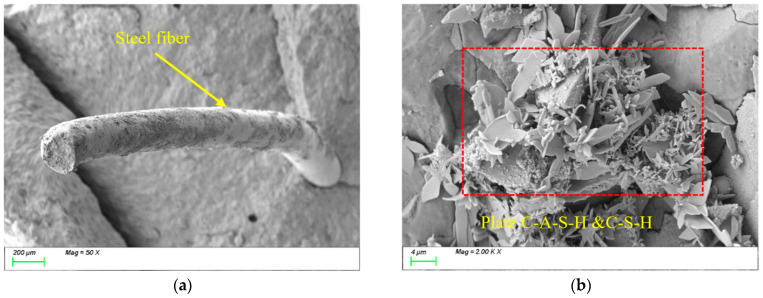
Microscopic image of ST0.5 before freeze-thaw cycle. (**a**) Steel fiber distribution; (**b**) characteristic product.

Figure 16 illustrates the evolution of microstructural morphology in ST0.5 specimens under varying freeze-thaw cycles. Figure 16a shows the SEM image before freeze-thaw cycles. It can be observed that the steel fibers are tightly connected to the matrix structure, with distinct physical bonding at the interface region. The steel fibers exhibit high surface roughness, which facilitates mechanical interlocking with the matrix. Additionally, their high modulus, high stiffness, and larger diameter effectively enhance the strength and toughness of the matrix. Figure 16b shows that the characteristic products within the matrix remain predominantly plate-like C-(A)-S-H gels, indicating that the incorporation of steel fibers does not alter the type or microscopic morphological characteristics of the characteristic products. However, the high modulus characteristics of the steel fibers inhibit crack propagation through the fiber bridging mechanism, thereby enhancing the overall mechanical performance of the material.

**Figure 17:**
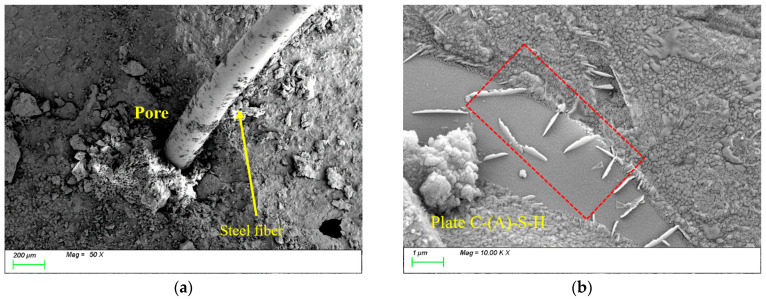
Microscopic image of ST0.5 after 100 freeze-thaw cycles. (**a**) Steel fiber distribution; (**b**) characteristic product.

After 100 freeze-thaw cycles, Figure 17a reveals the presence of voids at the interface transition zone (ITZ) between the steel fibers and the matrix, reducing the compactness of the matrix. Meanwhile, Figure 17b demonstrates that the internal structure of the matrix remains dense, with plate-like gel products tightly bonded to the matrix. This is primarily attributed to the subsequent reactions of the mineral powder during the freeze-thaw process, where newly generated products fill the matrix interior, thereby enhancing its compactness.

**Figure 18:**
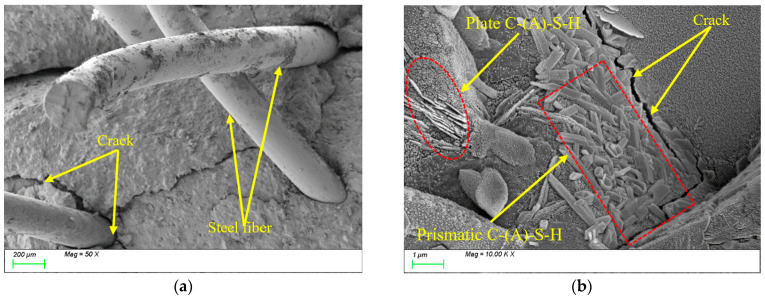
Microscopic image of ST0.5 after 400 freeze-thaw cycles. (**a**) Steel fiber distribution; (**b**) characteristic product.

After 400 freeze-thaw cycles, Figure 18a demonstrates that the deterioration at the interface transition zone (ITZ) between the steel fibers and the matrix has further intensified, manifested by cracks that have further expanded and formed interconnected structures. This results in reduced interfacial bonding performance between the fibers and the matrix. Meanwhile, Figure 18b reveals that the characteristic products within the matrix have developed more extensively, with abundant plate-like and prismatic products exhibiting interwoven growth. However, the formation zones of different characteristic products remain relatively concentrated, displaying an uneven distribution pattern. Longer cracks can be seen appearing inside the matrix.

In summary, although the incorporation of steel fibers does not alter the types of characteristic hydration products such as C-(A)-S-H gels during freeze-thaw cycles, their high stiffness modifies the filling mechanism of these products within the matrix from “free packing” to “restricted packing.” This transition influences both the reaction kinetics and spatial distribution patterns of the products, which may potentially compromise the “self-healing” effectiveness of the material system. Additionally, under the effect of temperature alternation, the significant difference in the coefficient of thermal expansion between steel fibers and the matrix leads to uneven deformation between them. This differential deformation, particularly at the interface, can induce crack formation. Therefore, it is noteworthy that although the internal structure of the matrix remains largely intact, damage in the interfacial transition zone (ITZ) remains a critical factor governing the overall matrix performance. Particularly during splitting tensile strength testing, interfacial defects are preferential pathways for crack initiation and propagation. This microstructural characteristic explains why the compressive strength loss rate of ST0.5 after freeze-thaw cycles was only 9.03%, while the splitting tensile strength loss rate reached 27.81%.

**Figure 19:**
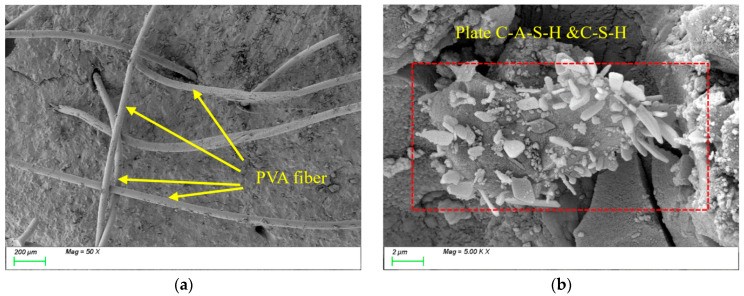
Microscopic image of PVA0.5 before freeze-thaw cycles. (**a**) Polyvinyl alcohol fiber distribution; (**b**) characteristic product.

Figure 19 illustrates the microstructural evolution characteristics of PVA0.5 specimens under freeze-thaw cycles. Figure 19a shows the SEM image before freeze-thaw exposure, revealing that randomly oriented PVA fibers within the matrix are interconnected, forming a three-dimensional spatial network structure. The flexibility and high ductility of PVA fibers enable effective bridging of microcracks at the microscale, significantly enhancing the material’s toughness. As shown in Figure 19b, the primary hydration products in the matrix are C-(A)-S-H gels, indicating that incorporating PVA fibers does not alter the types or micromorphological features of characteristic hydration products. However, the fiber bridging mechanism effectively suppresses crack propagation, improving the material’s mechanical performance.

**Figure 20:**
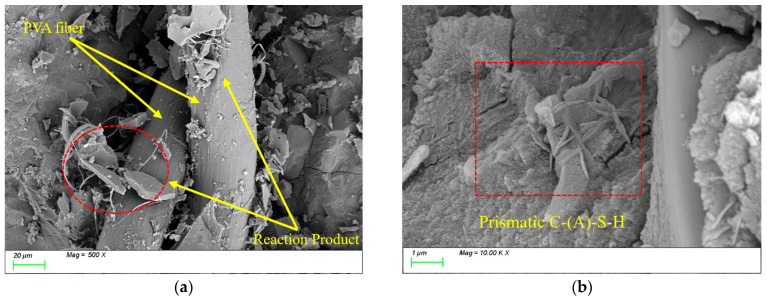
Microscopic image of PVA0.5 after 100 freeze-thaw cycles. (**a**) Polyvinyl alcohol fiber distribution; (**b**) characteristic product.

After 100 freeze-thaw cycles, Figure 20a reveals a substantial accumulation of hydration products on the surface of PVA fibers and their surrounding areas. These products exhibited two distinct morphological characteristics: one type displayed plate-like formations with well-defined edges and smooth surfaces, while the other manifested as acicular structures arranged in radial patterns. Energy-dispersive X-ray spectroscopy (EDS) analysis confirmed that these products primarily consisted of C-(A)-S-H gel alongside a minor ettringite phase (AFt). Meanwhile, Figure 20b demonstrates significant densification of the internal matrix structure, where prismatic and plate-like C-(A)-S-H products intertwine to form a continuous three-dimensional network. Similar to the observations with steel fibers, this phenomenon is primarily attributed to the subsequent reactions of mineral admixtures during freeze-thaw cycles. The newly formed products fill the internal matrix, thereby enhancing its compactness.

**Figure 21:**
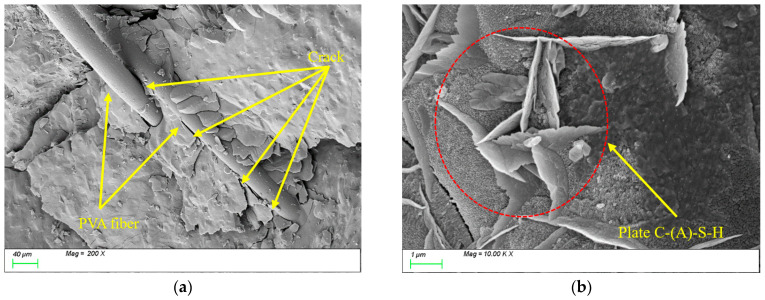
Microscopic image of PVA0.5 after 400 freeze-thaw cycles. (**a**) Polyvinyl alcohol fiber distribution; (**b**) characteristic product.

After 400 freeze-thaw cycles, Figure 21a reveals that under the continuous action of freeze-thaw cycles, distinct microcracks emerged at the interface between PVA fibers and the matrix, reducing the interfacial bonding performance. Meanwhile, as shown in Figure 21b, similar to the ST0.5 group, the characteristic products within the matrix underwent further substantial growth. A large number of plate-like products became embedded within the matrix, and the distribution of these products exhibited a certain degree of inhomogeneity. However, the difference lies in the distinct zonal distribution characteristics observed within the matrix interior, with no significant pores or interconnected microcracks detected. This is primarily attributed to the high elastic deformation of polyvinyl alcohol (PVA) fibers, which demonstrates negligible interference with expansive product filling while effectively alleviating expansion stresses caused by incompatible product filling and thermal deformation. Consequently, compared to the ST0.5 group, the PVA0.5 specimen exhibited compressive strength and splitting tensile strength loss rates of only 6.18% and 21.79%, respectively, after 400 freeze-thaw cycles, notably lower than those of the ST0.5 group.

## 5. Conclusions

This study evaluated the impact of mineral admixtures and fibers on the frost resistance of slag-yellow river sediment geopolymers, including compressive strength, splitting tensile strength, characteristic products, pore structure, and matrix microstructure. The following are the key findings of the study.

(1) The incorporation of silica fume and metakaolin significantly enhances the frost resistance of slag-Yellow River sediment geopolymers. Silica fume promotes secondary hydration reactions through its highly reactive SiO_2_, generating low calcium-to-silica ratio C-(A)-S-H gels that refine pore structures, resulting in a 19.9% increase in compressive strength after 400 freeze-thaw cycles. Metakaolin reinforces matrix stability by forming a three-dimensional N-A-S-H network structure, effectively delaying freeze-thaw damage, with a tensile strength loss rate of only 2.51% following 400 freeze-thaw cycles.
(2) The incorporation of fibers significantly influences the freeze-thaw performance of the material. Leveraging its high elasticity and deformation capacity, PVA fibers effectively mitigate frost heave stress and suppress microcrack propagation, with compressive strength and splitting tensile strength loss rates (6.18% and 21.79%) notably lower than those of steel fibers (9.03% and 27.81%). Due to their significant thermal expansion coefficient mismatch with the matrix, steel fibers are prone to interfacial crack formation under thermal cycling, thereby weakening the reinforcement effect.
(3) During freeze-thaw cycling, the material’s pore structure exhibits a two-stage evolution characterized by “refinement followed by coarsening.” In the early stages of freeze-thaw cycles, pore refinement is primarily driven by the post-reaction of mineral admixtures filling pores, leading to an increased proportion of gel pores, reduced macropores, and enhanced mechanical strength. In the late stages of freeze-thaw cycles, due to the continuous effects of temperature alternation and ice expansion stress, microcrack propagation occurs, which reduces the compactness of the matrix and causes a rebound in the proportion of macropores. This ultimately leads to the deterioration of mechanical properties at the macroscopic scale, with splitting tensile strength exhibiting more pronounced degradation.
(4) Through a five-dimensional analysis, it is evident that SF20 exhibits excellent durability in freeze-thaw environments. On the one hand, it demonstrates the lowest strength loss rate. It maintains a stable pore structure while also showing balanced performance in mass loss rate and relative dynamic elastic modulus. These properties make it suitable for frequent freeze-thaw environments such as road foundations and underground engineering. Additionally, this study provides a feasible solution for the resource utilization of Yellow River silty fine sand and industrial solid waste, offering both environmental and economic benefits.
(5) This study found that although the incorporation of fibers does not alter the types of characteristic hydration products such as C-(A)-S-H and N-(A)-S-H gels during freeze-thaw cycles, it significantly influences their reaction processes and spatial distribution patterns, thereby exerting a pronounced impact on matrix durability. This effect primarily depends on three critical factors: (1) the distribution of fibers within the matrix; (2) physical characteristic parameters of fibers, including thermal expansion coefficient, diameter, and aspect ratio; and (3) hydrophilicity and chemical adsorption effects. However, constrained by current experimental sample sizes and environmental simulation conditions, the long-term interaction mechanisms and practical implications require further in-depth investigation.

## Figures and Tables

**Table 1:** Chemical compositions of YRS and mineral admixtures (wt.%).

Minerals	SiO_2_	CaO	Al_2_O_3_	Fe_2_O_3_	K_2_O	TiO_2_	MgO	Other
YRS	68.64	8.40	12.33	3.25	2.55	0.74	2.05	2.04
Slag	32.47	41.06	14.52	0.28	0.44	1.25	7.08	2.9
SF	98.86	0.42	0.78	0.06	0.77	—	0.35	0.76
MK	53.57	—	44.40	0.94	0.73	0.19	0.09	0.08

**Table 2:** Performance Specification of Fiber.

Fiber Type	Diameter (mm)	Density (g/cm^−3^)	Length (mm)	Tensile Strength (MPa)	Elasticity (GPa)	Elongation (%)
ST	0.22	7.9	13	2800	210	5
PVA	0.04	1.3	12	1560	41	6.5

**Table 3:** The chemical composition of sodium silicate.

SiO_2_/(%)	Na_2_O/(%)	H_2_O/(%)	Density/(g/cm^3^)	Modulus	Beaume
30	13.5	56.5	1.51	2.3	50

**Table 4:** Mix proportion for a durability test.

No.	Sand	NaOH	SS	GGBFS	SF	MK	ST	PVA	Water
REF	1.000	0.020	0.128	0.660	—	—	—	—	0.192
SF20	1.000	0.020	0.128	0.528	0.132	—	—	—	0.192
MK20	1.000	0.020	0.128	0.528	—	0.132	—	—	0.192
ST0.5	1.000	0.020	0.128	0.660	—	—	0.032	—	0.192
PVA0.5	1.000	0.020	0.128	0.660	—	—	—	0.006	0.192

**Table 5:** Grouping of the frost resistance, mechanical, and microstructural property tests.

Properties	Performance Index	Specimen Size	Quantity
Frost resistance	mass loss rate	100 mm × 100 mm × 400 mm	15
	dynamic elastic modulus	100 mm × 100 mm × 400 mm	15
	compressive strength	100 mm × 100 mm × 100 mm	120
	splitting tensile strength	100 mm × 100 mm × 100 mm	120
Characteristic products	Thermogravimetric analysis	40 mm × 40 mm × 40 mm	45
	X-ray diffraction analysis	40 mm × 40 mm × 40 mm	45
Microstructural properties	porosity	40 mm × 40 mm × 40 mm	45
	scanning electron microscopy	40 mm × 40 mm × 40 mm	45

## Data Availability

The original contributions presented in the study are included in the article; further inquiries can be directed to the corresponding author.

## References

[1] Liu S., Liu C.Y., Hao Y.F., Zhang Y., Chen L., Li Z. (2024). Experimental investigation of engineered geopolymer composite for structural strengthening against blast loads. Def. Technol..

[2] Yang J., Wang Z.Q., He X.Y., Su Y., Tang Y.Z., Qi H.H., Yang C., Xiong G.Q. (2024). Using superabsorbent polymer to mitigate the fast setting and high autogenous shrinkage of carbide slag and sodium silicate activated ultrafine GGBS based composites. Sustain. Chem. Pharm..

[3] Cao B.S., Li Y., Li P.P. (2024). Synergistic Effect of Blended Precursors and Silica Fume on Strength and High Temperature Resistance of Geopolymer. Materials.

[4] Yang N., Xuan Q.D., Fu Y., Ma X., Lei D.Y., Niu J.L., Dai J.G. (2024). Phosphate activated geopolymer-based coating with high temperature resistance for sub-ambient radiative cooling Phosphate activated geopolymer-based coating with high temperature resistance for sub-ambient radiative cooling. Sustain. Cities Soc..

[5] Wang Z.K., Fu C.H., Wang K., Zhao J., Shumuye E.D., Yang Z.H. (2024). Effect of geopolymer concrete cover on improving tensile and transverse shear behaviors of BFRP bars after exposure to high temperature. Case Stud. Constr. Mater..

[6] Chen Z.M., Liu H., Zhu P.H., Li H.C., Ge T.Z., Yang L., Chen C.H., Dong Y.L. (2024). Effect of Curing Mechanism on Sulfuric Acid Corrosion Resistance of Geopolymer Recycled Aggregate Concrete. Ksce J. Civ. Eng..

[7] Zhou Y.W., Yu Y., Guo W.H., Xing F., Guo M.H. (2024). Development of inorganic anticorrosive coatings for steel bars: Corrosion resistance testing and design. Cem. Concr. Compos..

[8] Cui L.J., Xiang T.F., Hu B.J., Lv Y.J., Rong H., Liu D.E., Zhang S.Q., Guo M.L., Lv Z., Chen D.P. (2024). Design of monolithic super hydrophobic concrete with excellent anti-corrosion and self-cleaning properties. Colloids Surf. A Physicochem. Eng. Asp..

[9] Alzeer M.I.M., MacKenzie K.J.D., Low I.-M., Dong Y. (2021). Chapter 5—Fiber composites of inorganic polymers (geopolymers) reinforced with natural fibers. Composite Materials.

[10] Krishna R.S., Shaikh F., Mishra J., Lazorenko G., Kasprzhitskii A. (2021). Mine tailings-based geopolymers: Properties, applications and industrial prospects. Ceram. Int..

[11] Nuaklong P., Worawatnalunart P., Jongvivatsakul P., Tangaramvong S., Pothisiri T., Likitlersuang S. (2021). Pre- and post-fire mechanical performances of high calcium fly ash geopolymer concrete containing granite waste. J. Build. Eng..

[12] Figiela B., Bak A., Hebda M., Korniejenko K. (2023). Eco-friendly production of foamed geopolymers based on mine waste. J. Achiev. Mater. Manuf. Eng..

[13] Duxson P., Provis J.L., Lukey G.C., Van Deventer J.S.J. (2007). The role of inorganic polymer technology in the development of green concrete. Cem. Concr. Res..

[14] Dong C.H., Li T., Zhang Y.M., Liu J. (2018). Damage process and performance of PVA fiber-reinforced alkali-activated slag mortar plate under bending. J. Southeast Univ. (Engl. Ed.).

[15] Zhang S.Z., He S., Ghiassi B., Breugel K.V., Ye G. (2023). Interface bonding properties of polyvinyl alcohol (PVA) fiber in alkali-activated slag/fly ash. Cem. Concr. Res..

[16] Sarker P.K., Haque R., Ramgolam K.V. (2013). Fracture behaviour of heat cured fly ash based geopolymer concrete. Mater. Des..

[17] Pan Z., Sanjayan J.G., Rangan B.V. (2011). Fracture properties of geopolymer paste and concrete. Mag. Concr..

[18] Thomas R.J., Peethamparan S. (2015). Alkali-activated concrete: Engineering propertie sand stress strain behavior. Constr. Build. Mater..

[19] Atis C.D., Bilim C., Celik O., Karahan O. (2009). Influence of activator on the strength and drying shrinkage of alkali-activated slag mortar. Constr. Build. Mater..

[20] Lee M., Kim K., Chung C.W., Kim W., Jeong Y., Lee J. (2023). Mechanical characterization of recycled-PET fiber reinforced mortar composites treated with nano-SiO_2_ and mixed with seawater. Constr. Build. Mater..

[21] Lee B.Y., Cho C.G., Lim H.J., Song J.K., Yang K.H., Li V.C. (2012). Strain hardening fiber-reinforced alkali-activated mortar—A feasibility study. Constr. Build. Mater..

[22] Natali A., Manzi S., Bignozzi M.C. (2011). Novel fiber-reinforced composite materials based on sustainable geopolymer matrix. Procedia Eng..

[23] Zhang Y., Wei S., Li Z., Zhou X., Eddie, Chau C. (2008). Impact properties of geopolymer based extrudates incorporated with fly ash and PVA short fiber. Constr. Build. Mater..

[24] Zhang Y., Wei S., Li Z., Zhou X. (2009). Geopolymer extruded composites with incorporated fly ash and polyvinyl alcohol short fiber. ACI Mater. J..

[25] Akturk B., Akca A.H., Kizilkanat A.B. (2020). Fracture response of fiber-reinforced sodium carbonate activated slag mortar. Constr. Build. Mater..

[26] Zhuang X.Y., Chen L., Komarneni S., Zhou C.H., Tong D.S., Yang H.M., Yu W.M., Wang H. (2016). Fly ash-based geopolymer: Clean production, properties and applications. J. Clean. Prod..

[27] Wang B.M., Li G.N., Han J.N., Zheng Y., Liu H., Song W.Z. (2017). Study on the properties of artificial flood-prevention stone made by Yellow River silt. Constr. Build. Mater..

[28] Song Y.B., Xu J.X., Xu X., Wang P. (2023). Alkali-activated preparation and frost resistance of artificial flood-prevention stone material. Hydro-Sci. Eng..

[29] Wang Z.K. (2023). Multi-Sized Yellow River Sediment Activation and Its Application Research. Master’s thesis.

[30] Raza A., Zhang J.J., Xu S.W., Umar M., Yuan C.F. (2024). Experimental analysis of frost resistance and failure models in engineered cementitious composites with the integration of Yellow River sand. Sci. Eng. Compos. Mater..

[31] (2024). Standard for Test Methods of Long-Term Performance and Durability of Concrete.

[32] Zhang Y.K., Raza A., Umar M., Chen Y., Yuan C.F. (2023). Study on Frost Resistance and Interface Bonding Performance Through the Integration of Recycled Brick Powder in Ultra-High-Performance Concrete for Structural Reinforcement. Materials.

[33] Razzak A., Memon B.A., Oad M., Raza A. (2020). Effects of height to diameter ratio on compressive strength of recycled aggregate concrete. QUEST Res. J..

[34] Yuan C.F., Zhang J.J., Raza A., Fu W.C. (2025). Residual and damage properties of recycled brick powder-UHPFRC after high temperature. Proc. Inst. Civ. Eng.-Constr. Mater..

[35] Huang B.T., Wu J., Yu J., Dai J.G., Leung C.K., Li V.C. (2021). Seawater sea-sand engineered/strain-hardening cementitious composites (ECC/SHCC): Assessment and modeling of crack characteristics. Cem. Concr. Res..

[36] Zhang G., Jiang E.H., Li K.P., Shi H.W., Chen C. (2025). Study on the Influence and Mechanism of Steel, Polyvinyl Alcohol, and Polyethylene Fibers on Slag–Yellow River Sediment Geopolymers. Polymers.

[37] Ibrahim M., Johari M.A.M., Rahman M.K., Maslehuddin M.K. (2017). Effect of alkaline activators and binder content on the properties of natural pozzolan-based alkali activated concrete. Constr. Build. Mater..

[38] Song P.P., Liu Y.Z., Kong L.J., Tang Z.Y., Sun G.W. (2025). Research on design and optimization for compositions of ultra-high-performance geopolymer concrete. J. Build. Eng..

[39] Yu S.W., Xia M., Sanjayan J., Yang L., Xiao J.Z., Du H.J. (2021). Microstructural characterization of 3D printed concrete. J. Build. Eng..

[40] Huang H.F., An M.Z., Wang Y., Yu Z.R., Ji W.Y. (2019). Effect of environmental thermal fatigue on concrete performance based on mesostructural and microstructural analyses. Constr. Build. Mater..

[41] An M.Z., Huang H.F., Wang Y., Zhao G.Y. (2020). Effect of thermal cycling on the properties of high-performance concrete: Microstructure and mechanism. Constr. Build. Mater..

